# Exploring the Experiences of Families Impacted by the 2022 Commercial Milk Formula (CMF) Food Insecurity Crisis in the United States of America: A Scoping Review

**DOI:** 10.1177/08903344261419346

**Published:** 2026-03-26

**Authors:** Emily Jansch, Marjorie Atchan, Maryam Bazargan

**Affiliations:** 1 University of Canberra, ACT, Australia

**Keywords:** breastfeeding, breastfeeding experience, commercial milk formula, food security, formula feeding, human milk, Infant feeding, infant formula, lactation, nutrition policy, relactation

## Abstract

**Background:**

In the United States, the sociocultural reliance on commercial milk formula is prevalent from birth. Published data reveals that many parents and caregivers commence early initiation of commercial milk formula-feeding earlier than global health recommendations. The unprecedented shortage of commercial milk formula in 2022 exposed inadequacies in the infant food system and prompted adverse infant feeding challenges for parents and caregivers.

**Research Aim:**

To examine the experiences of families affected by the 2022 commercial milk formula food insecurity crisis in the United States.

**Method:**

The scoping review was guided by the Joanna Briggs Institute scoping review methodology and six databases were searched: CINAHL, MEDLINE, Scopus, ProQuest, and Google Scholar, and the Cochrane Database. This process generated 265 results with 174 full-text papers reviewed, and 18 articles retained for synthesis. Extracted data were examined following Braun and Clarke's thematic analysis framework.

**Results:**

Four themes were constructed: feelings of failure, vulnerable supply chains, making the most of availability, and lack of support for breastfeeding and relactation. The lack of support for relactation and breastfeeding was a recurrent issue for parents and caregivers. Commercial milk formula scarcity led to its adaptation with potentially significant health risks for these infants.

**Conclusion:**

The fragility of infant nutrition and food security in a commercial milk formula-reliant culture has been highlighted. The importance of national emergency preparedness and government policies that support parents and caregivers to have equitable and reliable access to commercial milk formula where indicated is demonstrated.

## Background

Optimal feeding from birth is defined as infants receiving breastmilk exclusively for the first 6 months of life; however, less than half of the world’s infants are exclusively breastfed to this age (World Health Organisation [WHO], 2021). Whilst it is important to acknowledge that for some infants with specific medical intolerances commercial milk formula (CMF) is a necessary dietary substitute, the recent increase in non-medically-indicated CMF feeding practices is characterised as a historically significant infant nutrition transition (Baker, 2020).

Whilst the influence of CMF feeding on maternal and infant health feature prolifically in published literature, consideration of the insecurity associated with a culture of reliance on infant CMF is minimal. Literature has explored infant feeding practices in low- and middle-income countries, with a focus on CMF utilisation (Baker et al., 2016; Neves et al., 2020; Walters et al., 2016), and examined its influence on breastfeeding (Rollins et al., 2016; Victora et al., 2016). The findings have reinforced the challenges unique to the political, sociocultural, environmental and economic conditions, including inadequate access to clean water, reduced or absent maternity leave, poor maternal nutrition, and practices unsupportive to breastfeeding.

CMF-feeding practices in high income countries are influenced by a combination of social, economic, cultural, and healthcare related factors including marketing and accessibility, cultural and social norms, workplace policies, perceived convenience and health benefits, and healthcare practices and recommendations (Baker et al., 2023; Neves et al., 2020). These conditions result in an increasing culture of reliance on CMF in high income countries where an estimated 21% of infants, never receive breastmilk (United Nations Children’s Fund [UNICEF], 2018). For example, published data reveals that many parents and caregivers in the United States commence early initiation of commercial milk formula-feeding despite global health recommendations (Centers for Disease Control and Prevention [CDC], 2022a). This may be associated with the supports available.

The health system in the United States is a complex, mixed-model combining private and public sectors. In comparison to other high-income counties, healthcare spending in the United States is the highest globally, yet disparities in access and outcomes persist (Schneider et al., 2021). Physiological infant feeding protections in the United States are less comprehensive than other high-income countries. Without universal paid maternity leave, and as lactation support requires insurance coverage, support for breastfeeding is fragmented and inequitable compared to more cohesive systems in other high-income countries (Feltner et al., 2018). This issue holds significant international relevance, as families worldwide rely on CMF, and it is plausible that similar shortages could occur in other countries. By moving beyond a solely quantitative description of outcomes, this scoping review provides insights that may inform practical strategies and policy responses applicable in diverse national settings, should comparable circumstances arise.

In 2022, the United States experienced a severe shortage of CMF as a result of global supply chain shortages precipitated by the COVID-19 pandemic. The U.S. government initiated regulations to ensure CMF-fed infants continued to have access to their food system given the disruption to supply chains and other food shortages (The White House, 2022). In February 2022, Abbott Nutrition ceased production of CMF at their Michigan plant following a Food and Drug Administration recall of several brands due to a *Cronobacter sakazakii* bacterial contamination that contributed to at least two infant deaths (CDC, 2022b). The domestic recall of CMF exacerbated global supply chain shortages leading to fluctuating and unpredictable nationwide out-of-stock rates of 18% to 90% (Jung et al., 2022; Paris, 2022).

Recent literature quantified the impact of CMF shortages during the COVID-19 pandemic (Barbosa-Leiker et al., 2021; Marino et al., 2023). The 2022 U.S. CMF shortage represents a standalone phenomenon as it demonstrates the potential vulnerability in the infrastructure of infant feeding in a high-income country. The aim of this study is to examine the experiences of families affected by the CMF food insecurity crisis in the United States.

**Key Messages**

• While the health impacts of commercial milk formula (CMF) feeding are well-documented, there is limited research on the insecurity linked to cultural reliance on formula—particularly in high-income countries where many infants never receive breastmilk.

• The study identified four main themes from the experiences of parents during the United States’ 2022 commercial milk formula shortage: feelings of failure, vulnerable supply chains, making the most of available resources, and lack of support for breastfeeding and relactation.

• This research reveals vulnerabilities in infant feeding infrastructure in high-income countries, and highlights the broad impact of CMF shortages on families. It emphasises the need for stronger support systems and better emergency response strategies for infant feeding.

## Methods

### Design

A scoping review was conducted to explore diverse literature sources reporting or describing parental experiences during the U.S. CMF shortage. While a narrow area of research, the emerging and current knowledge appears to be fragmented. Consequently, specific topic boundaries were required to facilitate focus and enhance relevance of findings. The rationale for choosing this method of review includes its capacity to determine key characteristics or factors related to a concept and to identify and analyse knowledge gaps (Munn et al., 2018). The existing knowledge base for food security for CMF-fed infants in high-income countries is scarce. Utilising the scoping review methodology enabled the researchers access to a greater breadth of resources than those found in a more traditional review. The systematic process embedded within a scoping review supports a thematic analysis that clarifies concepts, identifies research gaps, and offers an overview of current knowledge to understand parental experiences of this phenomenon. It lays the foundation for further research. Additionally, scoping reviews allow iterative refinement of inclusion criteria as understanding of the topic deepens (Mak & Thomas, 2022). This adaptability was key to a holistic capture of relevant information which was an effective tool in identifying and organising key themes of this emerging topic.

Guided by the Joanna Briggs Institute (JBI) protocol (Peters et al., 2020), the review systematically mapped existing studies and papers, following a transparent and replicable process. This approach has been used successfully in similar contexts to explore the complexity of factors that influence the changing dynamics of food security (Louie et al., 2022; Yii et al., 2020). The Braun and Clarke (2014) thematic analysis framework was utilised to ensure a comprehensive and nuanced understanding of the data. This flexible analytic approach of identifying patterns, themes, and gaps in the existing literature provided a clear and detailed overview of the research landscape complementing the broad and exploratory nature of a scoping review.

### Sample: Defining the Articles Reviewed

In constructing the research question, the PCC mnemonic framework was employed as recommended by the JBI protocol (Peters et al., 2020). For this mnemonic, P represents Population, C represents Concept, and C represents Context. Our research question was: What are the experiences of parents and caregivers of CMF-fed infants during the CMF shortage in the United States? (P: parents and caregivers of CMF-fed infants; C: experiences of the CMF shortage; C: United States of America, February 2022–December 2023).

Consultation with a Health Information Specialist librarian supported the development of the research question through an assessment of the scope of existing research. The primary researcher collaborated with the librarian in the development of a comprehensive search strategy, which included selecting appropriate databases and defining inclusion and exclusion criteria (Mak & Thomas, 2022). The question, criteria, and search strategy were then discussed with the team to reach consensus. The research inclusion and exclusion criteria are summarised in Table 1.

**Table 1.** Inclusion and Exclusion Criteria.

Parameter	Inclusion	Exclusion
Language	Published in English	
Context	United States	
Time Frame	January 2022–December 2023	Published prior to 2022 Published in 2024
Type	Peer-reviewed research, qualitative and mixed methods studies, grey literature	
*Grey-Literature	Published by a Government source or a national-level NGO. Published by national news media with a readership of > 1 million people. Includes stories or experiences of parents/caregivers who were affected by CMF shortages. Keywords as per the PCC mnemonic.	Unpublished or draft copies Published outside the United States Social media posts Podcasts and visual media

*Note.* NGO = non governmental organisation; CMF = commercial milk formula; PCC = Population, Concept, Context.

**Figure 1.**
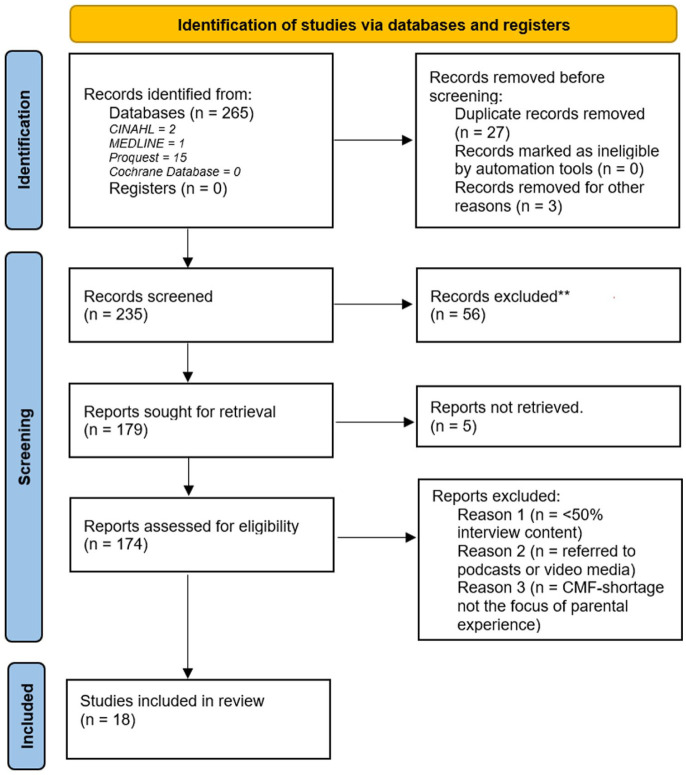
PRISMA 2020 flow diagram (Page et al., 2021).

Articles were excluded with less than 50% interview-to-story content to maintain focus on the experiences of participants. The PRISMA diagram describing the identification and screening process undertaken to reach the final sample for analysis is shown in Figure 1.

### Data Collection: The Search Strategy and Process

Data collection was conducted in January 2024 for resources published between February 2022 and December 2023. A systematic literature search was conducted in CINAHL, MEDLINE, Scopus, ProQuest, and Google Scholar, and the Cochrane Database. The PCC search string included key subject terms and their related themes “infant feeding,” “infant formula,” “commercial milk formula,” “baby,” and “newborn,” as well as the domains “shortage*,” “impact*,” “experience,” “security,” “insecurity,” and “food supply.” Publications that used non-cisnormative language including “chestfeeding” or “birth-parent” were used and remained eligible for inclusion due to their identity-affirming description of personal experience as a focus of the search strategy. Citation searching was employed to screen for additional relevant research. A general search for grey literature was performed utilising keywords and parameters within the domains of the PCC mnemonic. The full electronic search strategy is included in the online Supplementary Material File 1).

## Measurement

Methodological variables were present across the included studies, including cross-sectional surveys examining feeding practices during the CMF shortage and qualitative interviews exploring parental experiences. When identifying these variables, it was assumed that the content was an accurate and reliable self-reporting of experiences by parents and caregivers. Simplification was attended through summarisation of diverse parental experiences into common themes for mapping and analysis.

The primary researcher conducted data collection using the previously discussed and agreed strategy, identifying 265 articles. Agreed inclusion criteria were applied to titles and abstracts to mitigate subjectivity and ensure consistent decision-making, with full texts read if abstracts were unavailable. Agreed iterative refinement of the inclusion criteria occurred as understanding of the topic deepened. A total of 18 articles focusing on parents' or caregivers' experiences of the CMF shortage were selected for review and are described in Table 2.

**Table 2.** Summary of Papers Included in the Scoping Review.

First Author, Date, & Source	Study Aim	Sample	Design	Citation
Cernioglo (2023) Peer reviewed	Explore infant feeding practices across the United States during the infant CMF shortage that may pose nutritional and safety concerns.	*n* = 99 Low-income families that participate in the Special Supplemental Nutrition Program for Women, Infants, and Children.	Cross-sectional survey (electronic)	Cernioglo and Smilowitz (2023)
Jackson (2022) Peer reviewed	Examine US mothers’ perceptions of human milk banks as a response to the infant CMF shortage.	*n* = 863	Cross-sectional study (closed and open-ended items via social media)	Jackson and Obeng (2022)
Kalaitzandonakes (2023) Peer reviewed	Determine the proportion of US consumers who sought CMF during the shortage and identify coping mechanisms they used. Secondary aim: explore if there is public support for CMF policies.	*n* = 1070	Cross-sectional survey	Kalaitzandonakes et al. (2023)
Sylvetsky (2023) Peer Reviewed	Investigate mothers’ experiences during the 2022 infant CMF shortage in the United States and the perceived impacts on infants’ diet and health.	*n* = 28 *Mothers of infants under 8 months old	Online survey via RedCap	Sylvetsky et al. (2023)
Butler (2023) Masters’ manuscript	Investigate how low-income mothers coped with the baby CMF shortage.	*n* = 6	Interviews as part of a broader study	Butler (2023)
Carrazana (2022) Grey literature	Experiences of CMF shortages in rural areas.	*n* = 4	News article – interviews	Carrazana (2022)
Chuck (2022) Grey literature	Experiences of CMF-feeding parents who are unable to breastfeed.	*n* = 3	News article – interviews	Chuck (2022)
Kohli (2022) Grey literature	Parents experience of the CMF shortage.	*n* = 3	News article – interviews	Kohli (2022)
Lammert (2022) Grey literature	Childcare staff experiences of the CMF shortage.	*n* = 2	News article – interviews	Lammert (2022)
Luterman (2022) Grey literature	Examine the use of social media in connecting parents during CMF shortage.	*n* = 2	News article – interviews	Luterman (2022)
Pearson (2022a) Grey literature	Re-lactation and shame regarding breastfeeding Loss of access to specialty CMF.	*n* = 4	News article – interviews	Pearson (2022a)
Pearson (2022b) Grey literature	Experiences of parents with preterm infants during the CMF shortage.	*n* = 2	News article – interviews	Pearson (2022b)
Sandoval (2022) Grey literature	Experiences of feeding specialty CMF and changes to diets prompted by CMF shortage.	*n* = 4	News article – interviews	Sandoval et al. (2022)
Schreiber (2023) Grey literature	Reflect on the ongoing impacts of the US CMF shortage a year on.	*n* = 1	News article – interview	Schreiber (2023)
Schreiber (2022) Grey literature	Baby CMF shortage highlights inequality in U.S. maternal support.	*n* = 2	News article – interviews	Schreiber (2022)
Sharp (2022) Grey literature	Explore how parents are feeding their infants during the CMF shortage.	*n* = 3	News article – interviews	Sharp (2022)
Villalpando (2022) Grey literature	Describe one mother’s experience of the CMF shortage alongside the advice provided by a pediatrician to help navigate the shortage.	*n* = 1	News article – interview	Villalpando and Contreras (2022)
White (2022) Grey literature	Parents experiences of donating to milk banks and receiving donated breastmilk during the CMF shortage.	*n* = 2	News article – interviews	White (2022)

*Note.* CMF = commercial milk formula.

Included articles were read in full by the whole team. The first researcher descriptively summarised the papers, describing the study purpose, population, methods, and primary themes. The summaries were reviewed by the second and third researchers (MA, MB). Each researcher reviewed the data extraction table to identify information related to the research question and to note commonalities between articles. Codes were generated inductively through line-by-line review and key concepts extracted from the data were grouped into broader thematic categories to facilitate analysis. These constructs were evaluated through an iterative coding process, informed by Braun and Clarke’s (2006) thematic analysis framework, ensuring that emerging categories remained grounded in the data. Decisions about grouping were guided by conceptual similarity, relevance to the research question, and frequency across articles, with regular team discussions used to enhance trustworthiness and reduce interpretive bias.

To organise the extracted qualitative data, a structured thematic matrix was developed that facilitated comparison of recurring patterns, divergences, and contextual nuances across diverse literature types, including peer-reviewed studies and grey literature. Articles were listed in rows, while columns captured core elements such as author/year, study type, population and thematic content relevant to the review objectives. Inductive coding was applied to construct themes. Themes were continuously refined through an iterative review process to ensure consistency and relevance. This approach facilitated cross-source comparison and helped ensure transparency in how qualitative evidence was synthesised and represented.

### Data Analysis

Included sources constituted journal papers (*n* = 4), newspaper articles (*n* = 13), and a Master’s Thesis (*n* = 1). The journals are peer-reviewed and focus on disciplines including paediatrics, public health, preventive care, and maternal and child health. All journals are internationally recognised and based in high-income countries, and explicitly accept qualitative research, indicating alignment with the methodological focus of this review.

The newspaper publications were sourced from U.S. national (*n* = 4), state-specific (*n* = 7) and independent, non-profit (*n* = 2). All 13 were consistent with the inclusion criteria of a readership of > 1 million people.

All included content was published between 2022 and 2023 and within the United States. One paper was in preprint but has since been peer-reviewed and published. Publications primarily featured parents of CMF-fed infants (*n* = 17, 94%), with one article focused on the experiences of childcare staff as caregivers. Sample sizes ranged from one to 1070. Most of the included papers did not identify a methodology, being classified as news articles with interviews (*n* = 15, 83%). Cross-sectional surveys presented as mixed methods research was the predominant methodology identified (*n* = 3, 17%) and these are summarised in Table 3. This scoping review demonstrated internal methodological congruence through employment of a systematic search strategy across relevant databases, use of predefined criteria to extract data on study characteristics and outcomes, and application of thematic analysis to identify common themes and gaps in the literature. Regular meetings were held to compare interpretations, and an audit trail was maintained to ensure transparency in analytical decisions and reduce the potential bias.

**Table 3.** Measured Variables and Validity of Included Peer-Reviewed Studies (*n* = 4).

First Author & Date	Variables Measured	Measuring Instruments	Reliability & Validity	Citation
Cernioglo (2023)	Infant and maternal demographics Unsafe infant feeding practices Infants typical diet during the most challenging time of the CMF shortage	Authors created an online survey in Qualtrics	McNemar Test Blind quality control Validation survey for incomplete responses	Cernioglo and Smilowitz (2023)
Jackson (2022)	Milk banking as a viable alternative Sociodemographic variables	Authors created an online survey in Qualtrics	Pearson’s chi-square test Hosmer–Lemeshow Goodness of Fit Test	Jackson and Obeng (2022)
Kalaitzandonakes (2023)	Coping mechanisms used: switching CMF brands, purchasing CMF online, use of donor breastmilk. Sociodemographic variables	Authors created an online survey in Qualtrics	Validity not addressed	Kalaitzandonakes et al. (2023)
Sylvetsky (2023)	Sociodemographic variables Infant feeding practices Sources of information and beliefs about infant feeding practices and causes of the CMF shortage	Authors created an online survey via RedCap	Validity not addressed	Sylvetsky et al. (2023)

*Note.* CMF = commercial milk formula.

Data were then extracted and charted to include study characteristics and, where reported, the reliability and validity procedures employed.

## Results

Four themes were identified that characterised parents' and caregivers' experience of the CMF-shortage: feelings of failure, vulnerable supply chains, making the most of availability, and lack of support for breastfeeding and relactation.

### Feelings of Failure

Failure, as seen in the struggles to adequately provide infant nutrition, underscored the overall parental experience of the CMF shortage. The theme is broad as it includes emotional, mental, and financial hardships, as well as intangible impacts. Adverse emotional and mental health impacts were universally reported and characterised by family’s experiences of anxiety, depression, fatigue, guilt, shame, and failure. Internalised feelings of “failed” parenting dominated experiences and were most commonly expressed by parents of newborn infants. “It’s heartbreaking. It makes you feel like a failure as a parent, like you’re failing your children because you can’t even feed them… it makes you feel helpless” (Chuck, 2022, para. 20).

Time and money spent (lost) sourcing CMF was also described. An inability to adequately provide required nutrition meant large amounts of time were spent online researching community reports of CMF restocks, negotiating sales on informal supply chains, as well physically traveling to stockist locations to purchase CMF (Jackson & Obeng, 2022; Pearson, 2022a; Schreiber, 2022; Sylvetsky et al., 2023). With quantity restrictions in place during the shortage, parents and caregivers often travelled to multiple locations to collect single units of CMF to stockpile whilst units were available. “My husband and I probably went to 50 stores within a 20-mile radius. We had family members who were driving from Phoenix to L.A., stopping in every Target and grocery store on the way trying to find it” (Sharp, 2022, para. 21).

A further common consensus amongst parents was the time spent sourcing CMF affected their parent–infant bonding, exacerbating the guilt already present from feeling they may be unable to adequately feed their infant (Carrazana, 2022; Sandoval et al., 2022; Sylvetsky et al., 2023; Villalpando & Contreras, 2022). Feelings of failure intensified when parents were unable to purchase their preferred type of CMF, instead having to risk paying for CMF their infant had not tried and may not tolerate. “It feels like we are playing Russian roulette with our baby’s formula, which as new parents, isn’t a good feeling, because we don’t know what he’s allergic to. …Continuously trying new formulas is really scary” (Chuck, 2022, para. 10).

Vulnerable families including Black, Hispanic, and Latino parents who relied on the Special Supplemental Nutrition Program for Women, Infants, and Children scheme, a federal assistance program providing supplemental food, nutrition education, and healthcare referrals to low-income families to improve health outcomes and reduce food insecurity (Butler, 2023; Carrazana, 2022; Kalaitzandonakes et al., 2023; Lammert, 2022; Schreiber, 2022), also reported struggles. The already present intangible struggles for these families, exacerbated by the CMF shortage, now included difficulty accessing transportation to obtain CMF or unexpected costs associated with travel, high likelihood of living in a densely populated or rural area facing increased competition for CMF, and lack of lactation support in their employment. Parents in this demographic expressed frustration towards the restrictive parameters of government assistance programs in the wake of a food security crisis.

Now I feel like the government should be doing more. I wouldn’t say government control, but more government aid, assistance, regulation, whatever. There are babies going hungry. Moms are wondering if their babies are going to eat today or not. America is a first-world country. We should not be in this position. (Luterman, 2022, para. 25)

### Vulnerable Supply Chains

Parents and caregivers reported that their traditional methods of purchasing of CMF had become unreliable and they sought creative solutions (Butler, 2023; Carrazana, 2022; Cernioglo & Smilowitz, 2023; Jackson & Obeng, 2022; Lammert, 2022; Luterman, 2022; Pearson, 2022b; Sandoval et al., 2022; Schreiber, 2023; Sharp, 2022; Sylvetsky et al., 2023; Villalpando & Contreras, 2022; White, 2022). For example, online parent groups were formed to track CMF restocks and supply. “We feel so helpless because of this shortage. The group is one way we can feel a little less helpless. We can help connect people” (Luterman, 2022, para.14).

Whilst some described these groups as “life-saving” (Lammert, 2022), the surge of traffic to social media resulted in unregulated CMF markets which capitalised on parents' and caregivers’ desperation, with offers to purchase sample CMF as well as opened and expired cans (Cernioglo & Smilowitz, 2023; Luterman, 2022). Experiences of using the social media supply chain resulted in some vulnerable parents and caregivers reporting being scammed through false information and price gouging, such as taking payment for non-existent CMF or reselling CMF donated for a fictional infant (Kohli, 2022; Luterman, 2022; Pearson, 2022a; Sandoval et al., 2022).

Two weeks ago, K-Rae Knowles, of Oregon, Ill., sent money to a stranger in exchange for cans of a specialty formula she needed for her 4-month-old son, Callan. The cans never came, she said, and the seller’s Facebook profile was deleted a few days later.” “It’s really heartbreaking that people are preying on this kind of shortage” (Sandoval et al., 2022, para. 34–35).

Parents developed a reliance on friends and family members to source CMF where they were affected by geographical, financial, and time-frame limitations (Butler, 2023; Chuck, 2022; Lammert, 2022; Luterman, 2022; Schreiber, 2023; Sharp, 2022; Sylvetsky et al., 2023), which either increased or lessened the vulnerability of their situation. Parents reported that time spent sourcing CMF affected their infant bonding and exacerbated the guilt already present from feeling they may be unable to feed their child.

I'm on formula groups on Facebook. I am calling or texting or sending emails to friends and family, asking them for help. They're sending me texts, “Is this the right thing to get?” “How much do you want?” “Oh, I'm only able to get this much.” (Sylvetsky et al., 2023, p.19)

The reliability of CMF supply was reported as a significant stressor that influenced parental emotional and mental health and a motivator to secure CMF online in lieu of physical store availability (Carrazana, 2022; Sandoval et al., 2022; Sylvetsky et al., 2023; Villalpando & Contreras, 2022). Online procurement was identified as an effective strategy for families who could afford to purchase a subscription model of CMF supply. This option limited the brand accessibility and subsequently resulted in paying excessively for available CMF as opposed to their government-subsidised brand or familiar preferred variety. “After each feeding in the middle of the night, I look online and see if I could find formula instead of going back to sleep” (Sylvetsky et al., 2023, p.19).

Other ways parents reported experiencing the CMF-shortage included procuring samples from doctors and paediatricians. One article stated the hospital was sending newborn infants home with CMF to “help with the gaps” (Sharp, 2022, para. 16). Similarly, childcare centres became “swap points” for CMF during the shortage, and parents were able to exchange cans of acquired CMF for preferred brands or those with desired nutritional compositions, such as iron-fortified, preterm formula, and allergy-sensitive.

Parents bring in extra formula as they find it at stores. From there, Kid's Kingdom sets it out for families to take as needed. … The staff is also browsing stores and letting parents know if they find the formula their infant needs” (Lammert, 2022, para. 18).

### Making the Most of Availability

Parents reported “adapting” and changing CMF to extend current stock and strategies to bridge gaps between resupplies (Cernioglo & Smilowitz, 2023; Jackson & Obeng, 2022; Kalaitzandonakes et al., 2023; Kohli, 2022; Lammert, 2022; Luterman, 2022; Pearson, 2022a; Sandoval et al., 2022; Sharp, 2022; Sylvetsky et al., 2023; Villalpando & Contreras, 2022). Of the 99 U.S. parents who responded to an online survey (Cernioglo & Smilowitz, 2023), many reported alterations in their usual practice with the use of at least one unsafe infant feeding practice statistically increasing from 8% before the shortage to 48.5% during it (*p* < 0.005). There was also a change in the source of CMF from before to during the shortage, in both U.S. (79% to 27%, *p* < 0.005) and imported brands (39% to 11%, *p* < 0.005), with parents seeking alternatives.

Two articles specified that some parents also diluted their supply of CMF with animal or human milk to improve the nutritional quality (Cernioglo & Smilowitz, 2023; Sharp, 2022). Cernioglo and Smilowitz (2023) reported that there was a statistical increase from 2% to 29% (*p* < 0.005) in the use of watered-down infant formula. Parents acknowledged that health warnings were received during the shortage that advised against the practice of diluting CMF; however, their actions were motivated by feelings of desperation, frustration, and anxiety. Despite these warnings, and while not statistically significant, it is still concerning that 21 (5.6%) of the 1070 parents who responded to an online national survey (Kalaitzandonakes et al., 2023), disclosed they still attempted to make CMF at home.

Parents and caregivers reported rationing CMF to extend their supply by further spacing infant feeds or substituting a CMF feed for other nutrition (Butler, 2023; Sandoval et al., 2022). Parents also expressed how limiting portions of CMF exacerbated parental guilt and often led to the early introduction of solid food or the increase of solid-to-CMF ratio of an infant’s diet (Sandoval et al., 2022; Sharp, 2022; Sylvetsky et al., 2023).

I probably stretched some of the limits: “there's this much left, okay, we're going to stretch it and go a little bit.” … I am probably pushing solids a little bit more than I would be normally, just because it would be great to not be so reliant on formula and get off it quicker than I might have otherwise. (Sylvetsky et al., 2023, p. 21)

Parents stated that they substituted CMF types when appropriate CMF was not accessible (Carrazana, 2022; Schreiber, 2023), and nearly half (44%) of the 45 participants who completed a survey prior to a qualitative interview about their experiences reported switching between brands or types of CMF during the shortage (Sylvetsky et al., 2023). Type substitution included giving “follow on” or Stage Two CMF to neonates or giving “standard” CMF to infants requiring medically directed specialty formula. Parents also revealed they created their own formula where CMF was unavailable and options to obtain CMF were exhausted (Jackson & Obeng, 2022; Kalaitzandonakes et al., 2023). “Lennix had severe allergic reactions to nine different dairy-based formulas, she broke out in rashes, cried constantly and threw up everything she ate” (Sandoval et al., 2022, para. 26).

Parents indicated that they attempted to source donor human milk as a CMF alternative, but encountered accessibility barriers including limitation of geographical location and ineligibility to receive donor milk due to healthy, term infant status (Cernioglo & Smilowitz, 2023; Jackson & Obeng, 2022; White, 2022). The use of banked donor milk in Cernioglo and Smilowitz’s (2023) study significantly increased from 2% to 28% (*p* < 0.005) and from 5% to 26% (*p* < 0.005) for the use of human milk from informal sharing. A total of 668 women (77.4%) of Jackson and Obeng’s (2022) 863 survey respondents also stated a belief that donor milk was a viable response to the CMF shortage crisis. Statistically, marital status (*p* = 0.010), education level (*p* < 0.001), and religious affiliation (*p* < 0.001) influenced participants’ perceptions.

The identified barriers included official milk banks requiring prescriptions to dispense donated human milk. The circulation of informal supply lines of donor human milk through online platforms and social media were also reported. Despite government health warnings of unregulated donor milk, parents utilised human donor milk as a preferred alternative to diluting CMF or substituting it with other nutrition such as cow’s milk or water.

I … came across a post on social media that said that she was looking for breastmilk supplement because her supply was low and so I said, “Sure, why not?” … The difference between taking a donation from someone you know versus a milk bank is the milk bank screens the milk, they do a lot of safety precautions, then they also will pasteurize the milk. (White, 2022, para. 10)

### Lack of Support for Breastfeeding and Relactation

Breastfeeding and relactation featured as an option, albeit with little support. Parents attempted to induce or reinitiate lactation and breastfeeding but encountered a significant lack of support to do so (Schreiber, 2022; Sylvetsky et al., 2023). The lack of availability of lactation support providers was exacerbated by reduced postpartum services and education in the wake of the pandemic (Pearson, 2022a). Lack of representation and gender inclusivity (Chuck, 2022) was an obstruction to human milk feeding which increased reluctance to engage in lactation services.

The perceived lack of support extended to government services and policies including short and often unpaid parental leave, which was not adjusted or amended during the shortage (Sharp, 2022). Parents able to express breastmilk experienced reduced pressure to have to source CMF and rely on CMF feeding during the shortage. However, the investment of time into breastfeeding, pumping, and maintaining supply was not conducive to maternity leave conditions which increased anxiety and frustration for parents.

Breastfeeding requires time, being able to sit with your baby, being able to bond with your baby and working on your milk supply, not being able to go back to work. … It’s not that easy for some of us, even if we want to. (Schreiber, 2022, para. 21)

## Discussion

This scoping review examined the experiences of families affected by the 2022 CMF food insecurity crisis in the United States. Research has been published regarding changes to infant feeding practices as a result of the CMF shortage, with a focus on breastfeeding rates (Imboden et al., 2023) and infant-feeding outcomes (Damian-Medina et al., 2024). Whilst both studies involved parent and caregiver perspectives and opinions, they did not centre understanding the broad experience of parents and caregivers affected by the CMF-shortage as the primary objective. This scoping review collated the views of over 2000 parents and caregivers directly affected by the CMF shortage to build an understanding of their experience. The data described heterogeneous experiences unique to individual circumstances. The findings indicate that the CMF shortage had profound and multiple effects on parents and caregivers: vulnerability resulting from unreliability of sourcing was evident and a lack of support for re-lactation and breastfeeding was a contributor to the experience of distress.

Whilst lack of support for breastfeeding and relactation was the least described experience, its absence has broader ramifications. Where described, the relationship between CMF-feeding and breastfeeding was underscored by a lack of support and societal understanding. Mothers and parents expressed a sense of shame related to not breastfeeding which was driven by societal expectations. Public pressure to “just breastfeed” perpetuated feelings of guilt where parents had chosen not to breastfeed, whilst parents who were unable to breastfeed expressed feelings of failure. Parents and caregivers who considered infant feeding alternatives in Marino and colleagues’ (2023) work on the impact of the pandemic on feeding practices also experienced a lack of societal understanding and challenges exacerbated by difficulty accessing services and support due to sudden increased demands and decreased postpartum support offerings affected by a cascade of congestion in healthcare services. Interestingly, in Jackson and Obeng’s (2022) study, married women expressed more reservations about milk banking compared to those who were single or never married. Participants with education beyond high school were progressively less likely to view milk banks as a viable alternative, indicating a potential association between higher educational attainment and increased scepticism toward milk sharing practices. Additionally, women who did not identify as religious or spiritual tended to express less support for milk banking compared to those with religious or spiritual affiliations. These findings suggest that personal belief systems shaped attitudes toward communal infant feeding solutions during the shortage. A recent qualitative systematic review of 41 papers examining the views and experiences of women, donors, recipient mothers, and healthcare professionals regarding human milk donation or sharing lends weight to this concept (Li et al., 2024). The widespread physiological and emotional benefits for mothers, families and children reported to be associated with donating and receiving breast milk was influenced by the significant positive or negative effect of family members’ opinions, community values and religion, and discomfort linked to underlying beliefs and ideology about breastmilk.

The “perfect storm” triggered by the COVID-19 pandemic and the resulting lockdown exposed countless weaknesses and alarming deficiencies in healthcare systems and their lactation services, exacerbating breastfeeding disparities and challenges (DeYoreo et al., 2022; Schindler-Ruwisch & Phillips, 2021). Whilst breastfeeding in the United States is promoted and recommended through public health campaigns, our findings support the expressed view of others that the widespread availability of CMF coupled with pervasive marketing and advertising strategies has a powerful influence on parental choices and perceptions about infant feeding (Baker, 2020; Rollins et al., 2016; Van Tulleken et al., 2020).

Additionally, the United States does not have a comprehensive national policy that addresses the provision of breastmilk donation and usage. Oversight and regulation are managed through a combination of state guidelines and professional organisations' standards; however, these guidelines are largely not legislated (Rose et al., 2022). Interestingly, Jackson and Obeng (2022) reported the belief that donor milk was a viable alternative to address the CMF shortage, and Cernioglo and Smilowitz (2023) observed a substantial increase in the number of parents sourcing breast milk through informal community sharing. However, only 8.2% (*n* = 31) of those who sought CMF utilised donor milk as a coping mechanism in Kalaitzandonakes and colleagues’ (2023) study. The sum of findings indicates that a heightened demand for human milk alternatives in times of crisis did not overcome previously held concerns regarding safety and regulation and the weight of public opinion. The absence of a unified national policy creates variability in access to and awareness of donor human milk, which has led to greater reliance on CMF as a primary food source for infants (Bai & Kuscin, 2021).

The pre-pandemic culture of reliance on CMF enabled the targeting of vulnerable populations during the CMF shortage. Paediatricians and healthcare institutions providing free CMF to “bridge gaps” instead of supporting physiology, alongside disruptions to lactation services further diminished breastfeeding self-efficacy (Beheshti et al., 2022; Marino et al., 2023).

Published evidence and global policy supports breastfeeding and relactation as a protective response in emergencies (Dall’Oglio et al., 2020; Gribble et al., 2019; WHO, 2021). Middle- and high-income countries may contain a limited number of healthcare workers qualified to support breastfeeding relactation in emergencies (Hwang et al., 2021). The experiences of diminished support for relactation documented in countries such as Iraq, Myanmar, and Ukraine (Burrell et al., 2020; Haidar et al., 2017; Rahimov et al., 2022) show a similarity to our findings. These emergencies occurred primarily in low- and middle-income countries and humanitarian settings, however the events in the United States show that high-income countries are not immune. A recent systematic review of interventions to support relactation during emergencies in middle-income countries determined that in contexts where exclusive breastfeeding rates are low or CMF consumption is common, relactation support is less socially accepted and considered more challenging (Amat Camacho et al., 2023). Further research opportunities could include an examination of emergency responses to infant feeding crises in middle- and high-income countries, and strategies to support lactation or relactation. This evidence could inform the development of local and national health policy guidelines and procedures.

Infants are particularly vulnerable during a crisis due to their immature immune systems, specific food and fluid needs, and reliance on others for care needs (Gribble et al., 2019). Infants need specific strategies to protect, promote, and support their health needs during emergencies. Our findings outline that infants affected by CMF scarcity were additionally affected by inappropriate CMF adaptation and substitution, with as yet unknown short- and long-term health outcomes. Further research in this field is warranted. Our findings also highlight that the issues experienced by parents' and caregivers were exacerbated by the lack of national support strategies, and reveal the need for action, moving forward, to mitigate the risk should a further emergency situation occur.

## Limitations

The scoping review methodology facilitated a broad analysis of available evidence with a focus on individual experience within the inclusion criteria. Selection bias may have occurred with only news publishers with a readership of over 1 million being eligible for inclusion. Smaller publications may have documented individual experiences unique to a particular area or culture that could have informed new perspectives on the CMF-shortage; however, they may not have been comprehensively captured. As only English language publications were selected for inclusion, multilingual papers that represent the wider population of U.S. parents and caregivers may not have been captured. Despite these limitations, the findings of this scoping review were synthesised from individual experience documented across diverse sources which provided a holistic view of CMF-feeding as a practice and the CMF-shortage as a lived experience.

## Conclusion

Parental experiences of the CMF-shortage in the United States in 2022 were characterised by emotional, mental, and financial hardships. They also highlighted the challenges that existed in establishing breastfeeding and initiating relactation in this context. It is important to acknowledge that while CMF is indicated for some infants with medical intolerances, widespread adoption of CMF as a primary feeding practice creates a pressure point that is easily and negatively manipulated in the event of a disruption. The review also highlights the fragility of infant nutrition in a CMF-reliant culture without appropriate government-led preparedness for an infant feeding emergency.

## Supplemental Material

sj-docx-1-jhl-10.1177_08903344261419346 – Supplemental material for Exploring the Experiences of Families Impacted by the 2022 Commercial Milk Formula (CMF) Food Insecurity Crisis in the United States of America: A Scoping Review Supplemental material, sj-docx-1-jhl-10.1177_08903344261419346 for Exploring the Experiences of Families Impacted by the 2022 Commercial Milk Formula (CMF) Food Insecurity Crisis in the United States of America: A Scoping Review by Emily Jansch, Marjorie Atchan and Maryam Bazargan in Journal of Human Lactation
