# Supplementary material for: Exploring the Experiences of Families Impacted by the 2022 Commercial Milk Formula (CMF) Food Insecurity Crisis in the United States of America: A Scoping Review
Source: J Hum Lact. 2026 Mar 26;42(2):444–56. doi: 10.1177/08903344261419346 (PMC13263471; doi:10.1177/08903344261419346)
Supplement: sj-docx-1-jhl-10.1177_08903344261419346 – Supplemental material for Exploring the Experiences of Families Impacted by the 2022 Commercial Milk Formula (CMF) Food Insecurity Crisis in the United States of America: A Scoping Review [file sj-docx-1-jhl-10.1177_08903344261419346.docx]

The following search strategy was adapted for individual databases determined by their specific syntax. Individual search terms were combined using Boolean operators (AND, OR) to ensure a comprehensive search strategy.

*Infant Feeding*

("infant feeding"[MeSH Terms] OR "infant feeding"[Title/Abstract] OR "feeding practices"[Title/Abstract])

*Infant Formula*

("infant formula"[MeSH Terms] OR "infant formula"[Title/Abstract] OR "formula feeding"[Title/Abstract] OR “baby formula”[Title/Abstract])

*Commercial Milk Formula*

("commercial milk formula"[Title/Abstract] OR "commercial infant formula"[Title/Abstract])

*Baby*

("infant"[MeSH Terms] OR "infant"[Title/Abstract] OR "baby"[Title/Abstract])

*Newborn*

("newborn"[MeSH Terms] OR "newborn"[Title/Abstract] OR "neonate"[Title/Abstract])

*Shortage*

("shortage"[Title/Abstract] OR "shortages"[Title/Abstract] OR "scarcity"[Title/Abstract])

*Impact*

("impact"[Title/Abstract] OR "impacts"[Title/Abstract] OR "effect"[Title/Abstract] OR "effects"[Title/Abstract])

*Experience*

("experience"[Title/Abstract] OR "experiences"[Title/Abstract] OR "perception"[Title/Abstract] OR "perceptions"[Title/Abstract])

*Security and Insecurity*

("security"[Title/Abstract] OR "insecurity"[Title/Abstract] OR "food security"[MeSH Terms] OR "food insecurity"[MeSH Terms])

*Food Supply*

("food supply"[MeSH Terms] OR "food supply"[Title/Abstract] OR "food availability"[Title/Abstract])
